# Engaging communities in collecting and using results from verbal autopsies for child deaths: an example from urban slums in Freetown, Sierra Leone

**DOI:** 10.7189/jogh.09.010419

**Published:** 2019-06

**Authors:** Jennifer Hutain, Henry B Perry, Alain K Koffi, Megan Christensen, Emily Cummings O'Connor, Sonnia-Magba Bu-Buakei Jabbi, Thomas T Samba, Reinhard Kaiser

**Affiliations:** 1 Concern Worldwide, Freetown, Sierra Leone; 2 Department of International Health, Johns Hopkins Bloomberg School of Public Health, Baltimore, Maryland, USA; 3 Concern Worldwide US, New York, New York, USA; 4 Independent Consultant and formerly Concern Worldwide, Freetown, Sierra Leone; 5 Demographic and Social Statistics Division, Statistics Sierra Leone; 6 District Health Management Team, Ministry of Health and Sanitation, Freetown, Sierra Leone; 7 Centers for Disease Control and Prevention (CDC), Freetown, Sierra Leone

## Abstract

**Background:**

Verbal autopsies (VAs) can provide important epidemiological information about the causes of child deaths. Though studies have been conducted to assess the validity of various types of VAs, the programmatic experience of engaging local communities in collecting and using VA has received little attention in the published literature. Concern Worldwide, an international non-governmental organization (NGO), in collaboration with the Ministry of Health and Sanitation (MOHS), has implemented a VA protocol in five urban slums of Freetown, Sierra Leone. This paper provides VA results and describes lessons learned from the VA process.

**Methods:**

Under-five child deaths were registered by Community Health Workers (CHWs) in five urban slums between 2014 and 2017, and a specially trained local clinician used a VA protocol to interview caretakers. Symptoms were analysed using InterVA-4 computerized algorithm, a probabilistic expert-driven model to determine the most likely cause of death. Themes in care-seeking were extracted from multiple-choice and open-ended questions. VAs were implemented in collaboration with the community and the results were shared with community stakeholders in participatory review meetings.

**Results:**

Main challenges included limitations in death notification and capacity to conduct VA for all notified deaths. A total of 215 VA were available for analysis. Among 79 neonatal deaths aged 0-27 days, the most common cause of death was neonatal pneumonia (55%); among 136 children deaths aged 1-4 years, the most common causes were malaria (56%) and pneumonia (41%). Key themes in care-seeking identified included use of traditional medicine (14% of deaths), absence of care-seeking (23% of deaths), and difficultly reaching the health facility (8% of deaths that occurred at home) during fatal illness.

**Conclusions:**

Conducting VAs as a collaborative process with communities is challenging but can provide valuable data that can be used for local-level decision-making. The findings have practical implications for engaging the community and CHWs in reducing the number of these preventable deaths through expanded efforts at prevention, early and appropriate treatment, and reduction of barriers to care-seeking. A functional end-to-end VA system can enhance meaningful routine vital events monitoring by community, national, and international stakeholders.

The majority of deaths in low- and lower-middle income countries are not registered in national civil registration and vital statistics (CRVS) systems or assigned a physician-certified cause of death [1].Timely and accurate data on mortality and cause of death are essential to global and national health planning and policy development at the national level. However, it is also important for these data to be obtained and used locally by district-level health program implementers, practitioners, and communities to guide local program activities. This information, when used appropriately, also serves as a critical epidemiological surveillance mechanism to thwart emerging epidemics and other health threats, thereby mitigating public health disasters and enhancing health system resiliency [2].

Health information that can inform communities about the rates and leading causes of death among children in their own local communities in high-mortality, resource-constrained settings is limited. In order to engage communities more fully in the process of addressing the leading causes of mortality in the local community, there is a need to collect this information and share it with the community, thereby enabling local health care providers, community members, and parents to participate more fully in reducing the number of deaths among children younger than five years of age (hereafter, referred to as children under-five) from readily preventable or treatable conditions.

VAs are questionnaires administered to the person nearest to the deceased during the time leading up to death. The completed questionnaire provides information related to the symptoms leading to death, care-seeking practices [3], and basic information about the deceased. The information can be analyzed by a physician or by a computer-assisted algorithm to deduce the most likely cause(s) of death [1]. Additionally, social autopsy information can be gathered about the context in which the death occurred. Results of social autopsy questions may help with the design and implementation of effective interventions in reducing mortality [3-6].

In collaboration with the Sierra Leone Ministry of Health and Sanitation (MOHS), Concern Worldwide implemented the child survival project “*Al Pikin fo Liv”* (All Children Should Live) from 2011-2017 in 10 densely populated urban slum communities of Freetown, Sierra Leone. These communities are characterized by severe poverty, over-crowding, low-quality housing, limited access to public services, and poor sanitation. The project aimed to reduce maternal, infant, and child morbidity by increasing quality of primary care services, increasing positive household behaviors, strengthening community and district capacity to plan and conduct health activieies, and advocating for improvements in related policy and coordination. As part of an operations research study on the effectiveness of a participatory community-based health information system (P-CBHIS) [7], a sub-component of the larger child survival project, VAs for deaths in children under-five were routinely conducted and results were shared with local stakeholders in five of the communities. The main purpose of using VA in intervention communities was to examine whether sharing of results would help motivate community leaders to take actions to reduce the deaths due to these causes, and whether these actions would be successful in reducing deaths among children under-five. The Verbal Autopsy Officer worked in collaboration with other project staff to present results from recent VAs in each community meeting, followed by facilitated discussion and an action-setting exercise.

This paper provides VA results and describes the experience and lessons learned from the VA process, to inform use of VA in similar community-based projects.

## METHODS

### Study setting and process of death identification

Between October 2015 and May 2017, a Verbal Autopsy Officer with a clinical background as a Community Health Officer conducted VAs in five urban slum communities in Freetown which had 28 513 women of reproductive age (WRA) and 34 043 children under-five [8]. Volunteer Community Health Workers (CHWs), who were selected and trained under the child survival project to conduct monthly visits of approximately 25-30 households in his or her community, gave health messaging, referred sick children to clinics, and identified pregnancies, births, and under-five deaths. For deaths, the CHWs used paper forms issued by the MOHS to record the name of the deceased, age at death, address, and name of mother. Each month from October 2015 through March 2017, project staff collected the registers and transferred this information into a project-managed vital events database.

The VA Officer located families of the deceased and conducted interviews. Two individuals served consecutively in this role; both had backgrounds as Community Health Officers. The first VA Officer was trained by conducting supervised mock interviews and then pilot interviews with community members, and then receiving solicited feedback from project staff. He worked part-time for eight months. The second VA Officer had previous experience conducting verbal autopsies and received additional training from the first VA Officer during an employment overlap of one month in May 2016. The second VA Officer worked fulltime for 14 months. To reduce recall bias, interviews were prioritized by date of death (first in, first out). Later, as the backlog of cases grew, and older cases became difficult to locate, cases that occurred in the previous six months were prioritized. Logistical considerations, such as travel time, weather conditions, and availability of the respondent also affected the timing and order in which the interviews were conducted.

Two project field staff, called Community Development Officers (CDOs), served as the primary link between the intervention communities and the project. Before VAs were conducted, CDOs held meetings with key community stakeholders, including the Health Management Committee (HMC) chairmen, the Ward Development Committee (WDC) members, community chiefs, health facility in-charges, and women’s group leaders. The CDOs introduced and described the purpose of the VA, the VA tool, and the process of data collection. Verbal consent to proceed with the VAs was obtained from these key local stakeholders in each intervention community.

**Figure 1:**
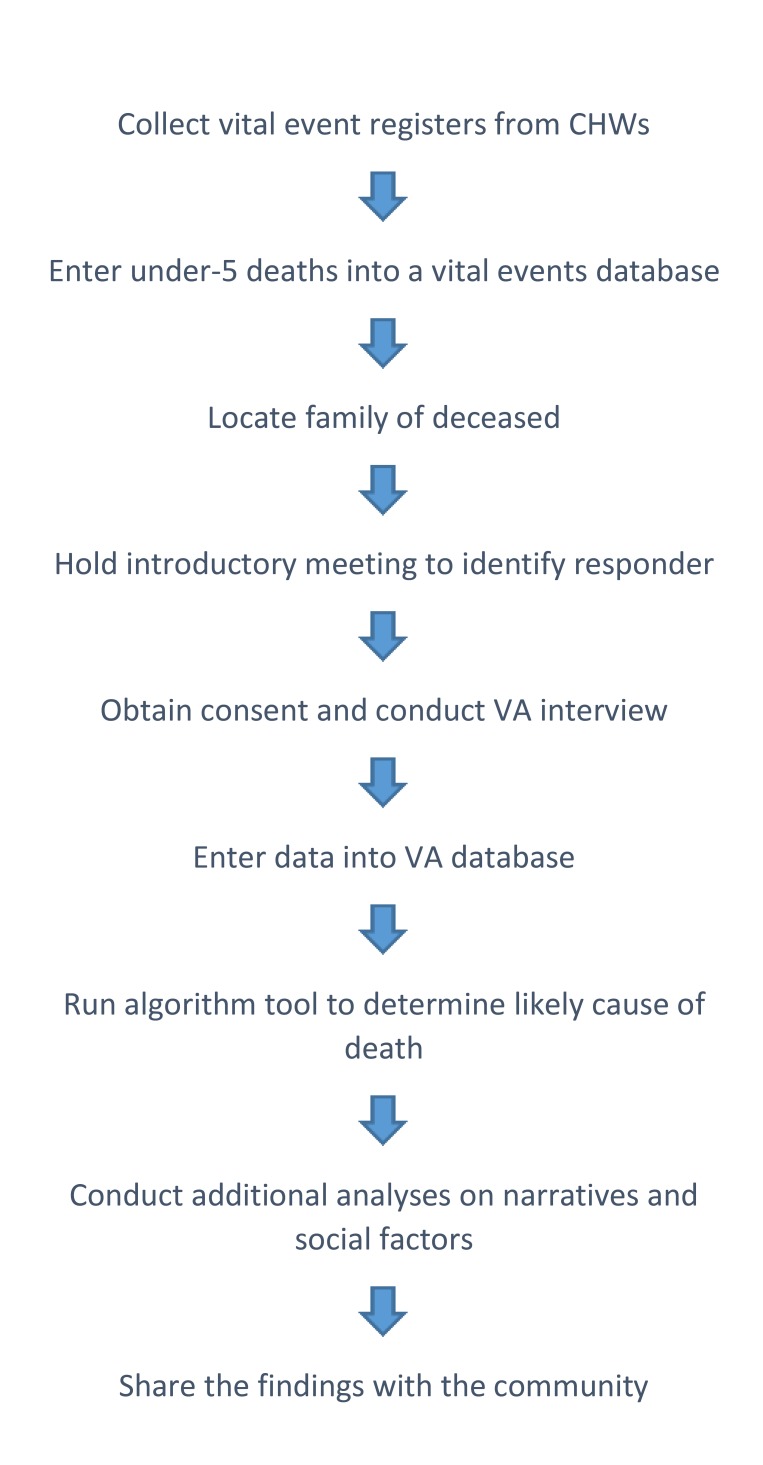
The basic steps used in conducting verbal autopsies for Concern Worldwide Child Survival Project Operations Research in Freetown, Sierra Leone, 2015-2017.

For each death, the VA Officer engaged the CDOs, the reporting CHW, and the CHW’s supervisor to help contact the family of the deceased and arrange for an introductory meeting. At the initial meeting, the VA Officer introduced himself, consoled the family, described the VA purpose and process, identified the most appropriate respondent, and arranged a time and place to conduct the VA interview. Consent from the respondent was obtained at the time of the interview. **Figure 1** gives highlights of the VA process.

The VA Officer interviewed the person who was the primary caregiver at the time of the child’s death. To increase the respondent’s comfort with the interview, it was common for a community leader – usually a member of the HMC or WDC—to attend the introductory meeting and the interview along with the CHW or CHW supervisor. No remuneration was provided to the respondent.

### Data collection instruments

We used the following VA instruments as they became available: (1) from October 2015 to February 2016, the WHO 2007 Verbal Autopsy Questionnaire [2]; (2) from June 2016 to July 2016, the Population Health Metrics Research Consortium (PHMRC) Shortened Questionnaire [9]; and (3) from August 2016 to April 2017, the WHO 2014 Verbal Autopsy Questionnaire [2]. Questions on all three instruments were similar and concerned the following topics: 1) demographic information, 2) symptoms of the illness(es) prior to death, 3) prior medical history, 4) health care received, and 5) a narrative description of events leading to death. For neonatal deaths (occurring during the first 28 days of life), questions about the mother’s health and the birth were also included. The VA protocol had additional questions related to the care-seeking activities of the family prior to death as well as attitudes and opinions of the caretakers, which together comprise the “social autopsy” elements of the assessment.

The survey questions were written in English and verbally translated by the VA Officer to Krio (the local language) at the time of the interview. The responses were recorded in English. Translations of key phrases and terminology, especially those describing symptoms, were available Krio (local language) alongside the English questionnaire text to help prompt the interviewer. For the first 11 months of data collection, VAs were conducted using a paper-based tool; but after that the data were entered into a digital tablet. Survey responses were entered (for paper forms) or uploaded (for digital forms) into an online platform called Ona [10] that collected and stored the responses.

### Analysis

We conducted a mixed-methods analysis of the VA results. We quantified specifications from the interviews including age of the deceased, duration of interview, and length of time between death and interview. Cause of death approximations were determined by a physician for the first 13 cases for the purpose of sharing with the community stakeholders. The three VA questionnaire types, including the initial 13 cases, were later mapped into the algorithm template. Causes of death were determined by the computerized probabilistic tool, InterVA-4 (version release 4.03 20160112) (Umeå Centre for Global Health Research, Umeå, Sweden, http://www.interva.net/) [11] chosen because of its open accessibility and ease of use.

After each indicator in the questionnaire was mapped for use in InterVA-4, responses were downloaded into Excel and converted for use in the tool. InterVA-4 uses an expert-driven probabilistic model to produce up to three causes of death with likelihood scores for each case. In cases resulting in more than one cause of death, the cause with the highest likelihood score was used in the results and analysis. Required parameters for the prevalence of malaria and HIV/AIDS in the study area were set to “very high” and “low,” respectively. When data were insufficient or contradicting, the probabilistic model returned an “indeterminate” result. Detailed descriptions of the method and interpretation of the tool’s results are described elsewhere [12,13]. Data were also downloaded into Excel for further qualitative coding and demographic disaggregation.

Project staff coded responses to open-ended explanations of events leading to death. The staff then analyzed these findings together with answers to single-choice, multiple-choice, and short-answer questions to identify important themes describing the context and care-seeking behaviors. For example, cases were determined to have sought care from a traditional healer if they had listed traditional healer as a source of care during the structured interview and/or mentioned seeking care from a traditional healer in the narrative description of the events leading to death. Case studies and data from the social autopsy components of the questionnaire were selected by program staff for presentation to CHW trainings and bi-monthly Community Health Data Review (CHDR) meetings attended by local community leaders and health center staff. Various methods were used to present the VA results but often the case study, de-identified, was read out loud and meeting attendees were asked to discuss why the death occurred and what, if anything, could have been done to prevent it.

### Ethical considerations

This study received Institutional Review Board (IRB) approval in February 2013 from the Sierra Leone Ethics and Scientific Review Committee. The study was declared exempt from review by the Johns Hopkins Bloomberg School of Public Health Institutional Review Board.

## RESULTS

Volunteer CHWs reported 582 deaths of children under-five in the intervention communities during the study period. 243 (41.8%) of these deaths were recorded in the vital events database and targeted for VA interviews. Verbal Autopsies were conducted for 222 deaths, which occurred from May 2014 to April 2017. All those contacted and requested to participate in a VA agreed to participate. The project staff were unable to locate some target respondents due to movement out of the community. Four VA interviews were conducted on cases with a recorded residence outside Western Area, where Freetown is located. InterVA-4 assigned a diagnosis of “stillbirth” to 7 cases, resulting in a denominator of 215 for analysis. Among those, one or more causes of death was assigned to 206 cases, and for 9 cases cause of death was indeterminate. Most of the respondents (96.4%) were a parent of the deceased. **Table 1** lists background characteristics of the deceased.

**Table 1:** Sociodemographic characteristics of 215 deceased children aged under-five with verbal autopsies, from five urban slum communities in Freetown, Sierra Leone, 2014-2017

Characteristic	Number (%)
**Sex:**	
Male	101 (47.0)
Female	114 (53.0)
**Age at death:**	
0-27 days	79 (36.7)
1-59 months	136 (63.3)
**Ethnicity:**	
Temne	100 (46.0)
Mende	15 (7.0)
Fulla	9 (4.2)
Limba	9 (4.2)
Other	24 (11.0)
Not recorded	58 (26.0)
Year of death:*	
2014, wet season	1 (0.5)
2014-2015, dry season	20 (9.3)
2015, wet season	50 (23.3)
2015-2016, dry season	83 (38.6)
2016, wet season	43 (20.0)
2016-2017, dry season	17 (7.9)
Unknown	1 (0.5)

*The wet season in Sierra Leone is May-October, dry season November-April.

Over one-third (36.7%) of the deaths occurred during the neonatal period. About half (47.0%) were males. There was no obvious concentration of deaths by season (wet vs dry). 75.8% of the children had been born in a hospital or health facility, 10.2% at home, 1.4% on route to hospital or other, and 12.6% did not know or did not answer the question.

The median length of the interview was 1 hour 17 minutes (IQR = 1:10-1:26) and the median time between death and interview was 7.7 months (IQR = 3.1-13.0).

### Cause of death

**Figure 2:**
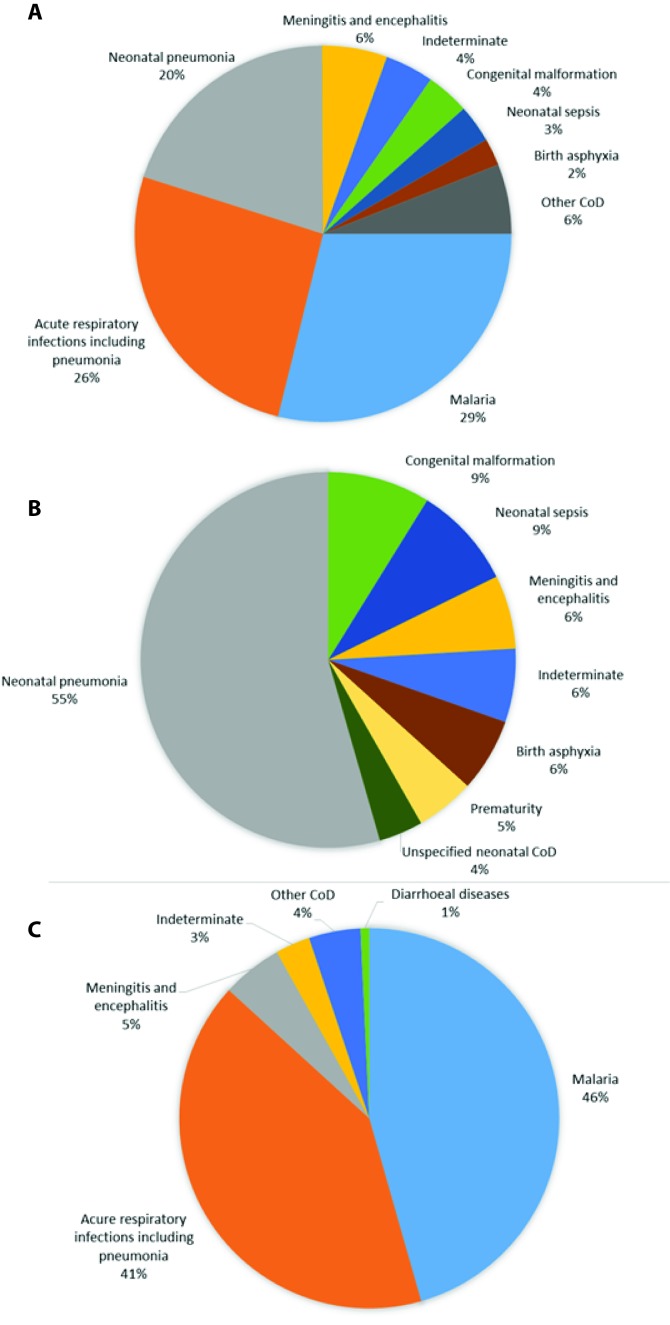
**Panel A.** Highest likelihood probabilistic-determined causes of deaths from verbal autopsies in 215 children who died before reaching five years of age, from five urban slum communities in Freetown, Sierra Leone, 2014-2017. **Panel B.** Highest likelihood probabilistic-determined causes of deaths from verbal autopsies in 79 deceased children aged 0-27 days, from five urban slum communities in Freetown, Sierra Leone, 2014-2017. **Panel C.** Highest likelihood probabilistic-determined causes of deaths from verbal autopsies in 136 deceased children aged 28 days to <5 years of age, from five urban slum communities in Freetown, Sierra Leone, 2014-2017.

The most likely causes of death for children under-five are shown in **Figure 2**, panel A-C. Acute respiratory infections, malaria and neonatal conditions were the most common causes of death in all children under-five. InterVA-4 assigned diarrhea as a cause of death for only one case (0.5%).

### Themes arising from a review of care-seeking behaviors

The behavioral, social, and health-system issues arising from this analysis are summarized in **Table 2**. In 14% of the cases respondents reported that they considered the cause of death to be supernatural or they sought care from a traditional provider. For nearly one-quarter of the deaths, there was no engagement with the formal health system, and 12% of the caretakers had doubts about the need for medical care for their sick child. A small percentage (4%) of those who obtained medical care reported mistreatment by the staff. In almost all (81%) of the cases in which the child died at the health facility, the caretaker reported the health staff did not inform the family of the cause of death. One-fifth (18%) of the caretakers who sought care did so from a private provider. There are some substantial differences between results for neonates and children 1-59 months regarding the health providers used and places of death.

**Table 2:** Themes in care-seeking derived from verbal autopsies in 215 deceased children aged under-five with, from five urban slum communities in Freetown, Sierra Leone, 2014-2017

Care-seeking behaviors	All (0-59 months)	Neonates (1-28 days)	Children (1-59 months)
**Had doubts about need for medical care**	**12.3% (20/163)**	**1.6% (1/63)**	**19.0% (19/100)**
Sought care from at least one provider	76.7% (165/215)	55.7% (44/79)	89.0% (121/136)
Of those, sought care from a private provider	17.0% (28/165)	11.4% (5/44)	19.0% (23/121)
Of those, sought care at private facility or location as first point of care*	12.7% (21/165)	11.4% (5/44)	13.2% (16/121)
Of those, reported mistreatment by health care staff*	4.2% (7/165)	4.5% (2/44)	4.1% (5/121)
Sought care from traditional provider and/or considered the cause of death to be spiritual/supernatural*	14.0% (30/215)	5.1% (4/79)	19.1% (26/136)
Of those, sought care from a health facility before traditional provider*	46.7% (14/30)	0% (0/4)	53.8% (14/26)
**Access to care:**			
**Died at home**	36.7% (79/215)	27.8% (22/79)	41.9% (57/136)
Of those, caretaker had difficulty reaching the health facility*	7.6% (6/79)	4.5% (1/22)	8.8% (5/57)
**Died at a health facility**	50.7% (109/215)	62.0% (49/79)	44.1% (60/136)
Of those, the health care worker did not inform caretaker of cause of death	80.7% (88/109)	87.8% (43/49)	78.3% (47/60)
Died en route to hospital or health facility, or other location	12.6% (27/215)	10.1% (8/79)	14.0% (19/136)
**Early neonatal deaths:**			
Died during the first week of life	21.9% (47/215)	59.5% (47/79)	N/A
Of those, born at home	27.7% (13/47)	27.7% (13/47)	N/A
Of those, born en route to hospital or health facility	2.1% (1/47)	2.1% (1/47)	N/A
Of those, born at a health facility	63.8% (30/47)	63.8% (30/47)	N/A
Of those, discharged early (within 48 hours of birth)*	26.7% (8/30)	26.7% (8/30)	N/A

*Based on an analysis of the open-ended narrative (in part or in whole).

### Sharing verbal autopsy findings with community stakeholders

After the first presentation of causes of death at a CHDR meeting in July 2016, the project staff learned that community stakeholders were particularly interested in the stories of families and the challenges they encountered in obtaining care for their child, as they were already aware that malaria and pneumonia were major killers of children in the area. The thematic findings from the VAs were presented for discussion in CHDR meetings in intervention communities from September 2016 through March 2017. Outcomes of sharing verbal autopsy and other data with community stakeholders are in the accompanying paper. Overall, communities conducting VAs and participating in bi-monthly meetings performed slightly better than those who did not in measurements of CHW functionality, household behaviours, and care-seeking behaviours [7].

“Normally, when someone is dead, maybe it is a result of the parent or the health worker. When you probe to get the information, it helps us to correct our mistakes for the future and make recommendation.” – Staff member of the Grey Bush Peripheral Health Unit, from focus group discussion, March 2017

## DISCUSSION

The cause-of-death results indicate that vulnerability for children under-five in these communities is highest in the first month of life. Predictably, malaria and pneumonia are the most common causes of death but we observe a lower-than-expected incidence of diarrheal diseases based on other reports on Sierra Leone [14-17]. Comparisons to other data sets are shown in **Table 3**.

**Table 3:** Comparison of verbal autopsy results from five urban slum communities in Freetown, Sierra Leone, 2014-2017, with other sources for top five causes of death for children under-5

	Top five causes of death for children under 5		
**Ranking**	**Freetown, Sierra Leone, 2014-2017**	**World Health Organization, 2015** [16]	**Institute for Health Metrics and Evaluation, 2016** [18]
1.	Malaria	Malaria	Malaria
2.	Acute respiratory infections including pneumonia and neonatal pneumonia	Acute lower respiratory infections	Lower respiratory infection
3.	Meningitis and encephalitis	Other communicable, perinatal and nutritional conditions	Diarrheal diseases
4.	Congenital malformation	Prematurity	Ischemic heart disease
5.	Neonatal sepsis	Diarrheal Diseases	Neonatal encephalopathy

The social autopsy results reveal challenges faced by caretakers of young children who are ill and living in urban slums. We observed that the formal health care system is not satisfying some of its patients who are then sometimes seeking care from traditional healers and other providers. Therefore, initiatives which encourage caretakers to utilize the formal health care system are not adequate to ensure appropriate care and treatment; interventions must also raise the standard of care at these facilities. We also observed some of the caretakers reported difficulty reaching the health facility, a result we did not expect in urban areas where at least 80% of the population lives within 2.5 km of a health facility [18]. However, people seeking care in these communities face non-distance barriers like darkness and security risks at night, and must make decisions about the value of health services against such risks. Some differences in results for neonates and children 1-59 months are substantial, however, we expect that circumstances for ill neonates are different than for older children. For example, neonates who died within days of birth may have never left the health facility in which they were born, inflating the numbers who “sought care” and decreasing opportunity to seek care with alternative providers.

Families and individuals living in Freetown commonly have very close ties to family living in the rural provinces of Sierra Leone. In some cases, children from rural areas are transported to an urban area for treatment of illnesses and conditions which are not readily addressed in their place of residence. Thus, some of the deaths included in our analysis could have been among children who were not long-term residents in the community. Future, more rigorous studies, might want to obtain more information on this point so that it would be possible to accurately determine the cause of death structure among those children who are permanent residents.

These results could be used at multiple levels to drive design and implementation of behavior change communication in the community, health facility trainings and improvements, and stakeholder decisions. These identified barriers relate not only to the health system directly, but also to the weak infrastructure of the informal communities; therefore, solutions require multi-sectoral engagement.

This study supports the notion that verbal autopsies can provide meaningful insights into important health issues for communities. Carrying out the VAs with a collaborative approach and sharing the results provided opportunities to increase community awareness of frequent deaths of young children in the community, the causes of these deaths, and the challenges that families encounter in obtaining treatment for a sick child. Prior evidence shows that the use of verbal autopsy data in health program planning can lead to improvements in health outcomes [13]. Most published reports of VAs have been carried out as large scale (often national-level) formal research studies in which interviewers were not part of the local communities and are not stakeholders in the health of the local population.

Involving community stakeholders in the entire VA process is paramount for the collaborative VA approach we have developed. Initial and informed approval from local stakeholders encourages acceptance of this sensitive activity in their community as well as ownership of the process and its results. CHWs and community stakeholders attended the interviews as well as the community meetings where the results were discussed. This increased the acceptance by the interviewed family members, and it provided stakeholders with an intimate understanding of the health-related issues faced by families whose children who are critically ill. This community involvement, coupled with sensitivity training for project staff, led to no refusals by family members of the deceased to participate. The process also put relevant information into the hands of those with some ability to effect change in their communities—through enactment of local policies, management and supervision of local health facilities and CHWs, and advocacy to regional and national government agencies.

While cause-of-death findings from verbal autopsies are useful for policy makers, practitioners, and other key decision makers, thematic social autopsy findings that are customized to the local context can be quite important to community stakeholders, particularly when common causes of death are widely known but contextual factors contributing to deaths are less well-known. The combination of social autopsy and verbal autopsy data can be used to generate awareness and advance understanding of specific health-system issues and social and behavioral issues that are specific to the particular context from which the data were obtained. Providing such information is empowering to communities and enhances their participation in the health system by engaging them in dialogue and action planning for how and where communities can promote change [3]. CHWs, local health facility staffs, and community leaders have the capacity to take action on this information by disseminating health messages, mobilizing the community, and engaging in advocacy.

### Important aspects of the verbal autopsy process

Successful implementation requires VA interviewers who are proficient in the technical skills of conducting the interview, such as proficiency in the local language, knowledge of basic medical terminology, and ability to use a digital tablet. The interviews, which are often emotionally difficult for caretakers, must be conducted by those with advanced skills in interpersonal communication, empathy, and patience. These skills are important not only for conducting individual interviews, but also for upholding trust and respect for the VA process in the community. Additional advantages include flexibility in arranging the time of VA interview, mobility to travel to and between communities, and capacity to work effectively with community leaders. The interviewers must also have appropriate supervision and technical support.

Any programs seeking to develop a VA system that engages the community should consult and collaborate closely with community leaders and local health providers as well as with the national health and statistics authorities and with others engaged in civil registration, vital events, and mortality tracking. The strong relationships developed by the project’s CDOs with the project communities also contributed to the success of the VA protocol. The VA Officers relied on CDOs to build trust and make connections to community leaders and CHWs, who in turn helped to locate, contact, and provide introductions to the respondents. VA interviews were usually attended by a trusted member of the community acquainted with the respondent, such as the CHW who originally reported the death or a member of the HMC. Not only did this practice decrease the respondent’s discomfort with the interview, but it also provided a unique opportunity for others in the community to better understand the experiences of these families.

There are several types of VA questionnaires and analysis methods available for use at present. With the growing global interest in mortality tracking, these tools will continue to undergo revisions and version upgrades. Tools vary greatly in accessibility, validity, technical difficulty of use, and application for particular purposes. At the outset of this study, we sought expert opinion regarding which VA tool to use. However, we had to change the VA to twice, once because the computerized algorithm did not exist and once because of the release by the World Health Organization of a widely verified instrument. Fortunately, the differences in the VA questionnaires were minimal.

A project intending to utilize VAs should carefully consider the operating context, the intended use of the results, and the tools available. The selected tool(s) should then be pilot tested end-to-end from identification of deaths to analysis of results and sharing of the results with the community. A reliable and timely local vital events registration system is a major asset for a community-engaged VA system.

### Limitations

Our findings are based on a non-random sample of 215 deaths of live-born children who died before reaching five years of age. We understand that the VA results may not accurately reflect the true situation in the study population. On average, only about 40% of CHWs submitted a monthly report during the study period, resulting in a highly incomplete register of under-5 deaths. Similar projects in which CHWs register deaths through routine home visits should be carried out where the CHWs are more highly motivated and better supervised.

There may be selection bias of cases based on the homes that CHWs visited (for example, homes that are closer to the main road). Despite continuous sensitization about the purpose of death reporting and use of the data, families and/or CHWs may have been less likely to report deaths with symptoms associated with Ebola virus disease such as bleeding, fever, vomiting, and diarrhea due to fear of investigation or fines during and following the Ebola outbreak in 2014-2016.

Some of the deaths reported by CHWs were never entered into the database due to delays in data collection (partially as a result of disruptions related to the Ebola outbreak), disorganized filing of the paper registers, and delays in entering the data from the CHW reports into the electronic vital events registration system. Thus, the overall process of entering notified deaths in the database remained slow and incomplete. Furthermore, data submitted by the CHWs were at times inaccurate or incomplete.

Unfortunately, we do not have sufficient data to be able to compare cases with and without VA. In addition, VAs were not conducted for all entries in the database due to a long backlog of cases and insufficient human resources. A larger team of VA interviewers (and better support, such as motorbikes and digital tablets) would have made it possible to obtained VAs for all registered deaths.

Since local languages in Sierra Leone are primarily spoken languages with limited written standards, only key phrases were translated, potentially resulting in inconsistencies in the way questions were asked. Instruments, including validation methods such as cognitive interviewing should be field-tested in the local language [19]. We recommend obtaining WHO guidance for training of VA interviewers in similar projects [20].

The WHO recommends conducting verbal autopsies within one year of the death [2]. While 72% of the VAs were conducted within one year of the death, 57 (28%) took place after one year or more and may have less accurate results due to recall bias. Also, three different VA instruments were used, which is not ideal for consistency of data collection, analysis, or interpretation. The tools used slightly different questions, which potentially could have resulted in differences in causes of death results if one of the other instruments had been used for the same case. We did not attempt to determine the effect of using different instruments, changing from paper versions to tablets, or the impact of missing data on the results.

While the InterVA-4 VA protocol has been shown to produce plausible causes of death for children under-five in other settings [13], there is also evidence that it does not perform as well as other VA protocols [20,21]. Our analysis showed considerable differences in the percentage of deaths among neonates from pneumonia (55%) when compared to 7% in national-level data [17]. In contrast, the percentage of deaths among all children younger than five years of age was only 0.5% while for national level data it is 10% [17]. Although these findings raise questions about the validity of the instrument and our results, further research will be needed to resolve this issue. The low percentage of deaths from diarrhea among our VAs, if accurate, could reflect the benefit of vigorous handwashing promotion during and following the Ebola outbreak. The precision of the percentages of the various causes of death in our data are not so critical for two reasons. First, the purpose of our paper is not to define what the causes of death are in our study population but to describe the process of how we engaged the community in the process of data collection and analysis. Second, for the purposes of communicating the leading causes of deaths to the community, the precise percentages are not as important as are what the leading causes are.

Thematic findings likely underreport the presence of barriers or constraints to appropriate care seeking or their significance because the narrative questions are open-ended and data are based on spontaneously mentioned barriers or constraints. The results we present here are further limited because they do not include any perspectives of health care workers or any information that might have been available from health facility clinical records.

Although the specific details regarding cause of death and care-seeking behaviors should be interpreted with caution as a result of the many limitations of our data, our findings strongly support the value of engaging communities in verbal autopsies as a useful way to raise awareness about the burden of disease and possible ways of prevention. VA results should inform further cause-of-death reviews and development of a strategic action plan for preventive and curative interventions at both the facility and community levels for reducing mortality from these causes.

The fact that only 40% of CHWs submitted monthly reports might seem to imply that this VA activity is not scalable or sustainable. However, the new volunteer CHW program that the government of Sierra Leone had introduced as this study began was definitely not an ideal cadre with which to try out this activity. Further studies would be helpful in settings where the CHW platform is much stronger.

### Broader implications

WHO and other United Nations agencies have set ambitious goals for civil registration and vital statistics (CRVS) by 2020. One of the goals that relates to community health systems is for 50% of deaths that occur in communities to have (1) a probable cause of death determined in real time and (2) vital events data collection systems designed to identify deaths that are representative of the local population. This target complements the Sustainable Development Goals (SDGs), which also set specific targets for each goal. The third SDG, Good Health and Well-Being, includes deliberate mandates for (1) increasing the capacity of national stakeholders to manage global health risks, monitor early warning signs and reduce risk; (2) improving access to health services; and (3) further reducing preventable mortality in children under five and maternal mortality [1].

VAs are a valuable complement to CRVS, particularly in settings where physician-certified cause of death is not feasible. At global and national levels, VA data are useful for estimating the disease-specific burden. However, when taken in isolation, VA data can be misleading, particularly in resource-constrained settings with weak health systems [22]. Research findings have demonstrated the value of verbal autopsies, but evidence is limited about scaling up verbal autopsies for routine national application apart from research studies. Evidence is also limited regarding how VA data can be feasibly and effectively integrated with CRVS in low-income settings. Finally, little is documented about the process and use of VA findings by communities for planning and decision-making. Local health data are paramount in order for communities to strengthen their own local health system and address their local disease burdens [23]. In a 2015 study, Biswas et al. determined that verbal autopsies discussed in local government health systems can contribute to improved health of mothers and newborns [24].

Verbal autopsy approaches and processes can be integrated in local contexts. They represent a significant opportunity for future operations research, especially when applied on a national, long-term, and sustainable basis. Evidence-based information will make meaningful contributions to strengthening health systems, particularly at a sub-national level. Experiences such as the one reported here can help refocus programs on the community as the cornerstone of the health system and a key decision maker for priority health problems [25].

## CONCLUSION

Conducting VAs as a collaborative, participatory process with communities generates valuable cause-of-death and care-seeking data that can be used for local-level decision-making. Findings have practical implications for engaging communities and CHWs in reducing the number of these (mostly) preventable deaths through expanded efforts at prevention and early treatment. A harmonized, community-level package that includes a standardized tool combining both verbal and social autopsy elements as well as a simplified, automated tool for data analysis is needed. Although there is growing recognition and appreciation for community participation in verbal and social autopsies, future products need to account for a two-way flow of timely information sharing that not only produces useful data for health system actors and policy makers, but also brings these stakeholders together with the community to generate meaningful dialogue and actions. End-user considerations should be taken into account for application in low resources settings with minimal technical support, significant logistical and capacity constraints and limited resources for scalability. Finally, a unified group of experts on verbal and social autopsies could bring together the international community as well as Ministries of Health to enhance CRVS and encourage wide-scale adoption of a standardized verbal and social autopsy tool.
